# Time, Not Sleep, Unbinds Contexts from Item Memory

**DOI:** 10.1371/journal.pone.0088307

**Published:** 2014-02-03

**Authors:** Roy Cox, Ron R. Tijdens, Martijn M. Meeter, Carly C. G. Sweegers, Lucia M. Talamini

**Affiliations:** 1 Department of Psychology, University of Amsterdam, Amsterdam, the Netherlands; 2 Amsterdam Brain and Cognition, University of Amsterdam, Amsterdam, the Netherlands; 3 Department of Cognitive Psychology, VU University Amsterdam, Amsterdam, the Netherlands; Imperial College London, United Kingdom

## Abstract

Contextual cues are known to benefit memory retrieval, but whether and how sleep affects this context effect remains unresolved. We manipulated contextual congruence during memory retrieval in human volunteers across 12 h and 24 h intervals beginning with either sleep or wakefulness. Our data suggest that whereas contextual cues lose their potency with time, sleep does not modulate this process. Furthermore, our results are consistent with the idea that sleep's beneficial effect on memory retention depends on the amount of waking time that has passed between encoding and sleep onset. The findings are discussed in the framework of competitive consolidation theory.

## Introduction

Contextual cues help us remember information that was learned previously in the same environmental context [1]. This so-called context effect is a robust finding in the memory literature, replicated in a host of situations [2]. Typically, subjects encode stimulus material in a certain physical environment and are later asked to recall that information in either the same environment, or a different one. At testing, congruence with the encoding context often results in better retrieval performance relative to testing in a non-matching or incongruent context. Contextual cueing can also take place on a more local scale, such as when individual items are presented with unique backgrounds that are either changed or kept constant at testing [3], [4]. Due to the limited overlap between distinct item-context pairs, such local cues are thought to produce more robust context effects than environmental cues, which remain constant for a large number of items [5].

Context effects are believed to be supported by the hippocampus, which, at encoding, binds spatially and temporally discontiguous features of an experience into an integrated episodic memory representation [6]–[8]. At retrieval, cueing with any component of the bound associative representation, including contextual components, will facilitate reinstatement of the whole encoded episodic representation.

While many studies have reported context effects on retrieval, few have considered extended retention intervals [9], [10], and these few show inconsistent results. However, several findings suggest that context effects might diminish over time, reflecting a change in the nature of memories as they age. Models of hippocampal memory processing suggest that configurational aspects of event memories (that is, the links between event components) should decay relatively quickly due to the high synaptic plasticity in this brain structure and the resultant fast overwriting of hippocampal representations [11], [12]. This process would lead to a decontextualization of memories over time, while sparing extra-hippocampally coded event components, such as individual objects. Indeed, a recent study supporting this notion showed that configurational aspects of a scene (object-object and object-location associations) are forgotten faster than object information [13]. Furthermore, studies in rodents showed that such decontextualization coincides with a diminished involvement of the hippocampus during retrieval [14], [15].

Interestingly, memory decontextualization occurs alongside system-level integration [16] and consolidation processes, whereby interference-prone hippocampus-dependent associations are recoded to a hippocampus-independent, more stable format [6], [17]. Sleep is of great importance for such memory consolidation [18], as evidenced by better memory performance after sleep relative to wakefulness [19], [20], reduced interference from competing information following sleep [21] (but see [22]), sleep-dependent shifts between neural retrieval networks [23], and correlations between memory retention and sleep architectural parameters or brain oscillations [24]–[26].

Despite accumulating evidence on the role of sleep in memory processes, its role in contextual memory has been little explored. (For some theoretically related accounts see [27], [28].) Of direct relevance, a recent report showed no difference in forgetting between sleep and wake conditions for items tested in a congruent environmental setting, but in incongruent surroundings memory was better after sleep than after wakefulness [29]. Hence, this study seemingly supports a decontextualizing effect of sleep. However, there was no reliable context effect at immediate testing, rendering the reported findings difficult to interpret. Two other studies claimed an opposite, strengthening effect of sleep on context. One considered a full night of sleep [30]; the other a short nap [25]. It should be noted, however, that the term ‘context’ in these studies refers to scenes that were explicitly associated with objects or nouns. Additionally, the retrieval tasks directly probed associative memory for these scenes. Therefore, these studies did not address the effects of implicitly encoded contexts on retrieval, but rather the effects of sleep on explicit associative memory.

In the present study, we assessed how time and sleep-wake patterns affect the influence of local, implicitly encoded contexts. We adapted a previously used memory task that manipulates contextual congruence at retrieval and shows a robust context effect [4]. Participants encoded nouns presented with unique, semantically unrelated, background photos. Later, they were required to complete word stem cues, half of which were presented with the same background as during encoding (congruent context), and the other half with a different one (incongruent context). Participants were assigned to one of four retention conditions, two with 12 h and two with 24 h intervals between two scheduled retrieval sessions (session I and II). The 12 h intervals entailed either day-time waking (W) or night-time sleep (S) between test sessions, while the 24 h intervals contained day-time waking followed by nocturnal sleep (WS) or the other way around (SW). This setup allowed us to investigate whether the context effect on retrieval diminishes over time, indicating memory decontextualization. Moreover, we could assess whether sleep and waking affect the context effect differently, while, through the 24 h groups, avoiding interpretation difficulties due to differing amounts of interference.

As contextual information was incidentally encoded in our task, we expected the formation of hippocampus-dependent noun-context links to be relatively weak. Competitive consolidation theories [31] suggest that during sleep, strong memories are preferentially reactivated and consolidated at the expense of weaker ones. Consequently, we predicted implicit contextual associations would not benefit much from consolidation processes during sleep. At the same time, the explicitly encoded word material may be expected to undergo sleep-related memory stabilization. Furthermore, and in line with studies showing memory decontextualization over time, we expected contextual cues to lose their efficacy at longer retention intervals.

## Materials and Methods

### Ethics statement

This study was conducted in accordance with the principles of the Declaration of Helsinki, procedures were approved by the University of Amsterdam, Department of Psychology ethics committee, and all participants provided written informed consent. Four participants under the age of 18 years also provided written informed consent themselves, as the ethics committee believed the experiment was not sufficiently burdensome or stressful to warrant additional consent from the next of kin.

### Subjects

A total of 108 volunteers agreed to participate in the study. After applying several exclusion criteria (initial memory score<30%; sleepiness score [see below]>4; sleeping less than 7 hours on the night prior to the experiment; sleeping outside the 11 PM-10 AM window in the 24 hours prior to, or during, the experiment; failure to comply with instructions regarding consumption of coffee, alcohol and psychoactive substances), 79 participants (64 female) with a mean age of 21.8 y (SD: 4.1; range: 16 – 37) were left for data analysis. All reported normal or corrected-to-normal vision, normal sleep-wake patterns and no history of neurological, psychiatric or sleep-related disorders.

### Procedure

Participants assigned to a condition starting with wakefulness (W [n = 19] and WS [n = 21]) reported at the laboratory between 8.30 and 11 AM. Similarly, subjects in conditions commencing with sleep (S [n = 19] and SW [n = 20]) arrived between 8.30 and 11 PM. Upon arrival, participants provided details regarding sleep in the previous night and recent psychoactive substance use. Sleepiness was assessed with the Stanford Sleepiness Scale (SSS). Next, instructions regarding the memory encoding task were given, followed by memory encoding and, after 1 min of retrieval instructions, subjects performed the immediate retrieval test (session I). Participants then left the laboratory and went about their regular business. After a period of either 12 h or 24 h (depending on condition, and allowing for a maximum deviation from the target interval of 1 h), they returned to the laboratory to fill out another SSS and perform the second retrieval test (session II).

**Figure 1:**
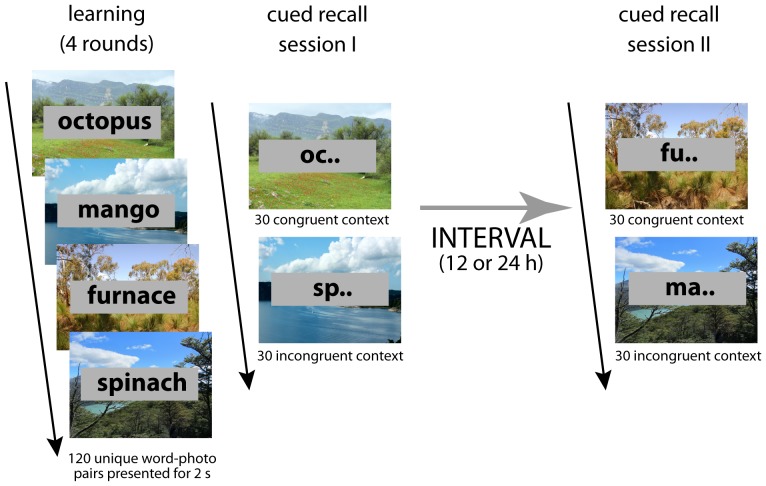
Schematic depiction of the memory task.

The memory task (Fig. 1) was presented using E-Prime 2.0 (Psychology Software Tools). It involved 120 common Dutch nouns, each beginning with a unique two-letter combination that was to serve as a cue at later retrieval. During learning, after a 1 s period displaying a central fixation cross, each word was presented for 2 s in a gray rectangle against a unique, unfamiliar background photo of a natural or city landscape. The landscapes were chosen not to contain individual distinguishing features (that is, buildings, hills, skies and vegetation were present in many photos, but none of these individual features were particularly distinctive or conspicuous). As such, the landscapes could not easily be recognized based on object characteristics alone; rather, it was the spatial layout of each landscape that contributed most importantly to its uniqueness. Subjects were asked to memorize the words, without any explicit instructions regarding the contextual photos. The full set of 120 word-photo pairs was presented four times in randomized order (± 20 min in total). For the two retrieval sessions, the encoding set was divided into two predetermined sets of 60 words. Word-set-to-retrieval-session allocation was counterbalanced across subjects. For each word, the corresponding two-letter word stem was presented in a gray rectangle against a background photograph. For both retrieval sets, half of the word cues (30) were presented with the same background photos as during encoding. For the other half, associated background photos from the encoding session were shuffled and re-paired with words into novel combinations. Allocation of items to the congruent and incongruent condition was randomized for each subject. During each retrieval session the word stem-photo pairings were shown in random order. Subjects were required to complete the word stem by typing the remaining letters of the corresponding studied word, without time restrictions.

### Data analysis

Following checks on normality all analyses were performed using parametric statistics. Per analysis, extreme outliers (>3 interquartile ranges below or above the first and third quartile, respectively) were removed (two data points in total). For individual tests the significance level was set at 0.05.

## Results

### Circadian influences

**Figure 2:**
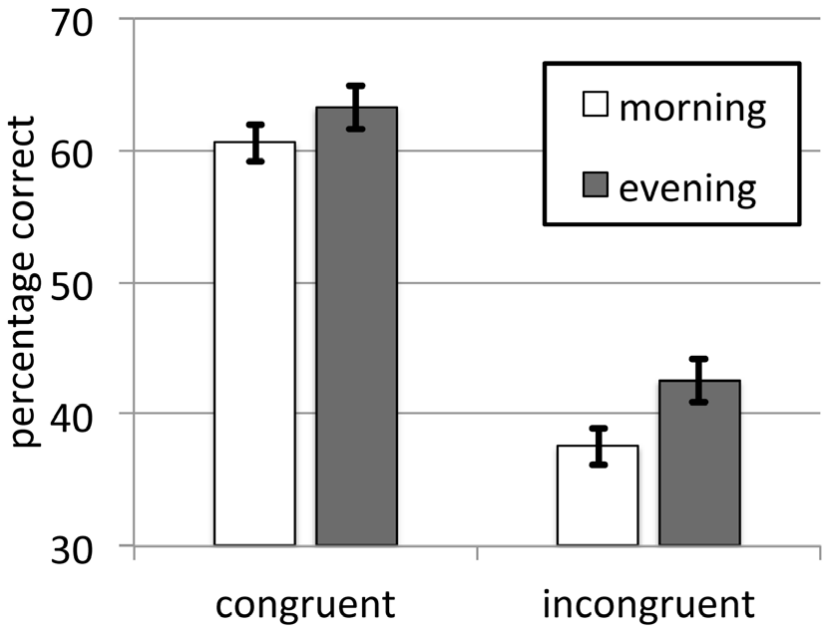
Memory performance (mean ± SEM) at session I for groups tested in the morning and evening. While there was a large context effect, no circadian effects were present.

First, to rule out any circadian influences on initial memory performance, we performed a 2×2 ANOVA with between-factor time of day (morning/evening) and within-factor context (congruent/incongruent) on session I memory scores (Fig. 2). For this analysis, 12 h and 24 h groups were combined. There was a highly significant positive effect of the congruent contextual cue on memory performance [F(1,77) = 100.9, P<0.001, η^2^ = 0.57], but no effect of time of day [F(1,77) = 2.0, P = 0.17, η^2^ = 0.03], nor an interaction between these factors [F(1,77) = 0.4, P = 0.53, η^2^ = 0.01]. Furthermore, subjective sleepiness at either retrieval session did not vary as a function of circadian phase (Mann-Whitney U, both P>0.3).

### 12 h interval

**Figure 3:**
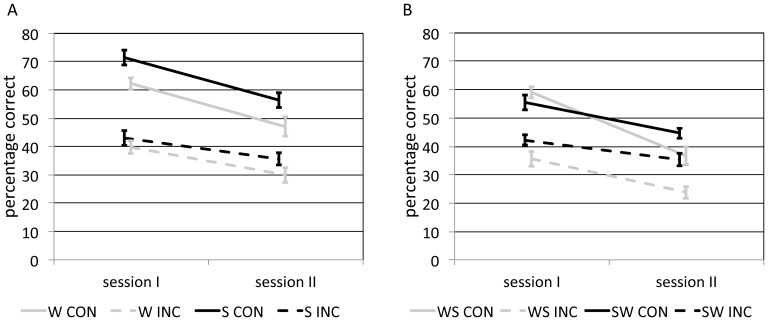
Memory performance (mean ± SEM) for 12 h (A) and 24 h intervals (B). W: wake, S: sleep, WS: wake-sleep, SW: sleep-wake, CON: congruent, INC: incongruent.

For the 12 h retention conditions (Fig. 3A, Table 1), we performed a 2×2×2 ANOVA with between-subject factor sleep-wake state (S/W), and within-subject factors context (congruent/incongruent) and session (I/II). As expected, congruent items were recalled significantly better than incongruent ones [F(1,36) = 56.6, P<0.001, η^2^ = 0.61] and retrieval scores were lower on session II than session I [F(1,36) = 67.1, P<0.001, η^2^ = 0.65]. In addition, there was no significant main effect of sleep-wake interval on retrieval [F(1,36) = 2.8, P = 0.11, η^2^ = 0.07]. Importantly, however, a significant interaction between session and context was found [F(1,36) = 4.1, P = 0.05, η^2^ = 0.10], indicating steeper forgetting for congruent than for incongruent items. All other interactions remained non-significant (all F<0.7, all P>0.20). These results suggest that retrieval’s initial benefit from contextual cues diminishes over time, independently of sleep-wake state.

**Table 1:** Memory performance for 12 h and 24 h groups (mean ± SD).

12h		session I	session II	24h		session I	session II
WAKE	congruent	62.3±16.3	47.0±23.0	WAKE - SLEEP	congruent	59.0±16.1	36.7±21.6
	incongruent	39.8±14.5	30.0±12.2		incongruent	35.6±12.7	23.8±9.9
SLEEP	congruent	71.4±19.4	56.3±21.7	SLEEP - WAKE	congruent	55.5±17.8	44.7±14.2
	incongruent	43.0±11.9	35.6±12.0		incongruent	42.2±13.0	35.3±14.9

### 24 h interval

A similar ANOVA carried out on the 24 h data confirmed significantly better memory for congruent than incongruent items [F(1,39) = 31.8, P<0.001, η^2^ = 0.45] and on the first relative to the second retrieval session [F(1,39) = 71.2, P<0.001, η^2^ = 0.65], with no significant main effect of sleep-wake order [F(1,39) = 2.6, P = 0.12, η^2^ = 0.06] (Fig. 3B, Table 1). As with the 12 h groups, there was a significant interaction, now more pronounced, between session and context, reflecting enhanced forgetting for congruent items [F(1,39) = 7.7, P = 0.009, η^2^ = 0.16]. Additionally, we found a significant interaction between session and sleep-wake order [F(1,39) = 7.2, P = 0.01, η^2^ = 0.16], indicating stronger forgetting for the WS group, regardless of contextual congruence. Other interactions were non-significant (both F<1.9, both P>0.20).

These results confirm the findings in the 12 h groups, showing that the benefit of contextual cues to memory performance diminishes over time, independent of wake-sleep state during the retention interval. Moreover, an overall retention advantage of sleeping shortly after encoding was revealed that is unrelated to contextual cueing.

### Controlling for potential confounds

In the analyses reported above, unequal performance levels for congruent and incongruent items at session I present a confounding factor that complicates interpretation of the results. In particular, enhanced forgetting for congruently cued items could be due to higher initial performance. Therefore, we calculated a proportional retention measure ([congruent session II/congruent session I] and [incongruent session II/incongruent session I]) and performed a paired t-test to compare proportional forgetting for the two context conditions. For this analysis, we pooled across all four sleep-wake conditions to compensate for the large increase in variance generated by the normalization. Retention was, again, significantly lower for congruent (mean ± SD: 72.9±24.7%) than for incongruent items [80.5±27.1%; t(76) = -2.0, P = 0.048], supporting the view of dwindling contextual assistance over time.

**Figure 4:**
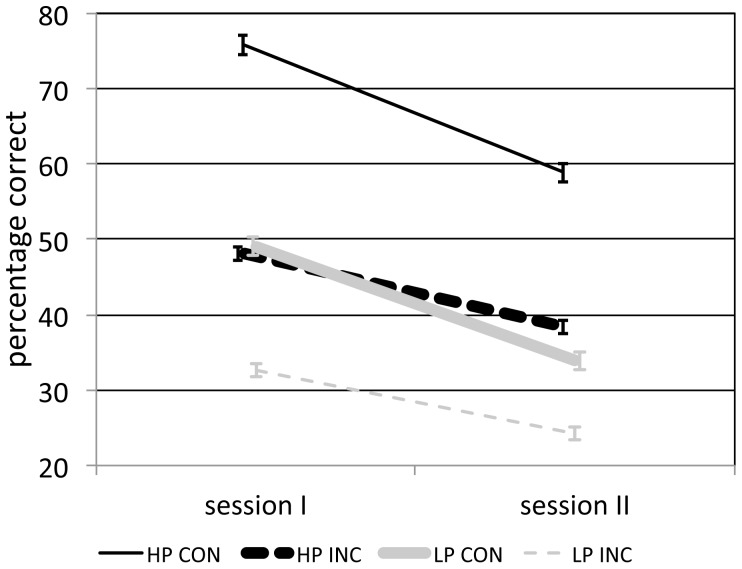
Memory performance (mean ± SEM) after performing median split to equalize initial memory scores for congruent and incongruent items. Congruent items in low-performing individuals (LP CON; thick solid gray line) are forgotten significantly more quickly than incongruent items in high-performing subjects (HP INC: thick dashed black line). For reference, congruent scores of high-performers (HP CON) and incongruent scores of low-performers (LP INC) are also plotted (thin lines). Statistics, however, were performed on the LP CON and HP INC groups (thick lines) only.

While the abovementioned approach normalizes performance with respect to session I scores, it is still based on values that are overall higher for the congruently cued items. In order to remedy this issue, we conducted a median split on the total score at session I (congruent score + incongruent score) across all four sleep-wake conditions, dividing subjects into low- and high-performers. We selected the incongruent scores of the high-performers, and the congruent scores of the low-performers, to achieve highly similar session I performance for both context conditions (Table 2). Indeed, an independent t test indicated these scores did not differ substantially (high-performers incongruent: 48.1±11.8%; low-performers congruent: 49.0±11.6%; t(77) =  –0.36, P = 0.72). Furthermore, this novel context factor was equally divided across all four sleep-wake groups [χ^2^(3) = 5.7, P = 0.13]. A subsequent 2×2 ANOVA with between-subject factor context (congruent/incongruent) and within-subject factor time (session I/II) was performed to test whether congruent items still decay faster than incongruent items when matched on initial performance. This analysis revealed a significant main effect of time [F(1,77) = 66.4, P<0.001, η^2^ = 0.46], and a significant interaction between time and context [F(1,77) = 3.3, P = 0.038, η^2^ = 0.04, one-sided], again confirming increased forgetting for context-congruent items (Fig. 4). The main effect of context was not significant [F(1,77) = 0.6, P = 0.43]. Importantly, we wished to ensure that increased forgetting of congruent items was not the result of overall greater forgetting in low-performing individuals. Thus, we compared just context-congruent, or just context-incongruent item retention between the two groups. The group-by-time interactions were not significant [both F(1,77) = 0.3, P = 0.3, one-sided], indicating high- and low-performers forget at similar rates. Thus, time-dependent decontextualization appears unrelated to differential initial memory performance in the congruent and incongruent context conditions.

**Table 2:** Memory performance after median split into low- and high-performers (mean ± SD).

		session I	session II
high-performers	congruent	75.8±12.8	58.9±21.4
	incongruent	48.1±11.8	38.4±12.9
low-performers	congruent	49.0±11.6	33.9±12.1
	incongruent	32.6±10.0	24.2±9.0

## Discussion

Our findings provide strong evidence that the enhancing effects of context cues on retrieval diminish over time, indicating memory decontextualization. Our results are inconsistent, however, with a specific role of sleep in this process. Furthermore, we found evidence that the order of sleeping and waking is of importance for retention, with superior memory when sleep follows shortly after learning, rather than after an extended period of waking.

Considering our main findings in more detail, we observed more extensive forgetting for congruent than for incongruent items, for both (12 h and 24 h) retention intervals. A likely explanation is that the links between words and their encoding contexts decay more quickly than the representations of the studied words themselves, decreasing the benefit of a congruent contextual cue with time since encoding. This account fits well with recent evidence that associational aspects of memory decay faster than object information [13]. Interestingly, the time course of decontextualization in the current study appears to be faster than in our previous report [13]. This may be due to the fact that incidentally encoded contextual links in the present study are probably a lot weaker than the explicitly encoded associative links in the Talamini and Gorree study [13]. As such, this difference may account for the differential forgetting rates. Our findings are also consistent with a few other studies showing a temporal decline of context effects on retrieval in humans [9], [10] and on conditioned responding in animals [14], [15].

While time appears to unbind contexts from item memory, this process does not appear to be linked differentially to the wake and sleep states. Indeed, there was no indication, either for the 12 h or the 24 h groups, that being awake or asleep affected the retention of congruent and incongruent items differently. Consequently, our results do not provide evidence for the hypothesis that sleep decontextualizes memories [29], nor do they support the idea that sleep consolidates contextual links [25], [30].

As noted earlier, the report of sleep-decontextualization by Cairney and associates [29] lacks an absolute context effect at initial testing, which hampers its interpretation. The apparent conflict between our results and the studies showing a benefit of sleep on contextual memory may be reconciled considering differences regarding the depth of encoding (intentional or unintentional). The Van der Helm [25] and Lewis [30] studies tested explicit associative memory, for which benefits of sleep have been reported fairly consistently [20], [21]. On the other hand, in our own study, contexts were incidentally, and therefore probably weakly, encoded. Neural network accounts of system-level consolidation suggest that weak hippocampal memory traces have a smaller probability of being reactivated and strengthened during sleep than stronger ones [31]. Thus, contextual links in our study may not have undergone notable system-level consolidation, because other, stronger representations in the network won the competition for access to consolidation processes. Specifically, consolidation may have favored explicitly encoded associations such as those among items or those between items and mental constructs used as a learning strategy.

Indeed, we did find an effect of sleep-wake order on overall memory, irrespective of contextual congruence. Specifically, night-time sleep followed by waking resulted in much better retention compared with day-time waking followed by sleep. This finding, too, may be explained in terms of competitive consolidation, considering that interference during waking potentially weakens memory traces. In the 24 h intervals, day-time interference and competitive sleep consolidation interact, such that sleep preferentially consolidates recent, and thus relatively strong, memory traces, which leads to enhanced subsequent waking retention, while day-time interference degrades representations that then benefit little from subsequent sleep. Hence, and possibly because of the longer retention interval, the effects of sleep and waking on retention appear more robustly over 24 h than over 12 h intervals. These results replicate previous findings in our lab [20]. More generally, they are in agreement with studies showing that the interval between learning and sleeping is of importance for subsequent retention, with better memory when sleep closely follows encoding [19], [32], [33].

Regarding the absence of a sleep effect in the 12 h interval, it may also be considered that our retrieval task, given the presence of a word stem cue, may rely somewhat less on hippocampal binding than pure associative tasks, for which sleep's benefit is usually more pronounced [20]. However, while intra-item pattern completion may indeed be served by extra-hippocampal cortices [34], our robust contextual effect on retrieval strongly suggests that, on the whole, our task was episodic in nature and hippocampus-dependent. We also wish to note that our use of unique, local visuospatial contexts with each item may be somewhat different from using a global background context that is relatively stable across episodes. However, in terms of the functional processes and neural circuitry involved, we presume local and global contexts behave similarly, as again suggested by the strong context effect.

Recently, it has been suggested that sleep helps decouple memories from their affective layer [35], but that this process requires several nights of sleep [36], [37]. By analogy, it is possible that differential effects of sleep and wakefulness on the (de)contextualization of neutral memory traces were not apparent in our study because we did not allow for the passage of sufficient time. However, this suggestion is based on findings relating to emotional aspects of memories, which may or may not hold for emotionally neutral visuospatial contexts like the ones we employed.

Limitations of the current study are that participants were not monitored in between memory retrieval sessions and may have encountered or rehearsed previously learned words. Similarly, because we did not track polysomnographic measures, sleep architecture could not be compared between groups and may have differed. Yet, we expect variability in these realms to have been distributed equally across groups. Considering possible time-of-day effects: While we did not assess psychomotor vigilance directly, subjective reports of sleepiness and comparable baseline performance for those tested in the morning and evening suggest circadian effects did not pose a major concern.

In interpreting our findings in the context of everyday life, it should be considered that, in our experimental setup, the amount of waking time (and thus interference) between learning and sleep onset was the most prominent factor determining differences in the representational strength of task items. However, in a real-life setting, both affective [32], [38] and motivational [39], [40] circumstances may importantly co-determine memory strength at sleep onset and subsequent sleep-related information reprocessing.

In summary, our results show how incidentally encoded contextual information loses its ability to trigger associated memory representations as time elapses. Furthermore, sleep does not contribute preferentially to this process. Nonetheless, sleep appears to stabilize memory representations that are relatively strong at sleep onset. All in all, the pattern of results across both the current and previous studies is most consistent with a competitive account of sleep-dependent memory consolidation.
